# Integration or Fragmentation of Health Care? Examining Policies and Politics in a Belgian Case Study

**DOI:** 10.34172/ijhpm.2021.58

**Published:** 2021-07-06

**Authors:** Monika Martens, Katrien Danhieux, Sara Van Belle, Edwin Wouters, Wim Van Damme, Roy Remmen, Sibyl Anthierens, Josefien Van Olmen

**Affiliations:** ^1^Department of Public Health, Institute of Tropical Medicine, Antwerp, Belgium.; ^2^Department of Family Medicine and Population Health (FAMPOP), Faculty of Medicine and Health Sciences, University of Antwerp, Antwerp, Belgium.; ^3^Centre for Population, Family & Health, Department of Social Sciences, University of Antwerp, Antwerp, Belgium.; ^4^Centre for Health Systems Research & Development, University of the Free State, Bloemfontein, South Africa.

**Keywords:** Stakeholder Analysis, Processual Analysis, Chronic Care, Integrated Care, Policy, Qualitative Research

## Abstract

**Background:** Globally, health systems have been struggling to cope with the increasing burden of chronic diseases and respond to associated patient needs. Integrated care (IC) for chronic diseases offers solutions, but implementing these new models requires multi-stakeholder action and integrated policies to address social, organisational, and financial barriers. Policy implementation for IC has been little studied, especially through a political lens. This paper examines how IC policies in Belgium were developed over the last decade and how stakeholders have played a role in these policies.

**Methods:** We used a case study design. After an exploratory document review, we selected three IC policies. We then interviewed 25 key stakeholders in the field of IC. The stakeholder analysis entailed a detailed mapping of the stakeholders’ power, position, and interest related to the three selected policies. Interview participants included policy-makers, civil servants (from ministry of health and health insurance), representatives of health professionals’ associations, academics, and patient organisations. Additionally, a processual analysis of IC policy processes (2007–2020) through literature review was used to frame the interviews by means of a chronic care policy timeline.

**Results:** In Belgium, a variety of policy initiatives have been developed in recent years both at central and decentralised levels. The power analysis and policy position maps exposed tensions between federal and federated governments in terms of overlapping competence, as well as the implications of the power shift from federal to federated levels as a consequence of the 2014 state reform.

**Conclusion:** The 2014 partial decentralisation of healthcare has created fragmentation of decisive power which undermines efforts towards IC. This political trend towards fragmentation is at odds with the need for IC. Further research is needed on how public health policy competences and reform durability of IC policies will evolve.

## Background

**Key Messages**

** Implications for policy makers**

- In a federal country, it is important that objectives and actions across different policy levels are aligned closely to improve policy coherence, accountability, coordination, and leadership in decision-making.
- Integrated care (IC) reform can only succeed if different forms of power (including technical, political, and financial resources) and leadership come together.
- To facilitate engagement and time investment of the health sector, financial stimulus is needed to move beyond voluntary commitment of health professionals and meso-level organisations (including patient and health professionals’ associations).
- There is a strong need for comprehensive follow-up and evaluation of policies, policy reform, and pilot projects to enhance political and organisational learning.

** Implications for the public**

This study examines the policy process and the influence of stakeholders on three integrated care (IC) policies of the last decade in Belgium. Our findings highlight stakeholders’ belief in the need for change towards a more patient-centred system, but also that this intention does not stroke with the current strongly provider-driven system and institutional design in Belgium. This study shows that Belgium’s fragmented political structure itself clashes with the IC paradigm. Belgium’s political structure is characterised by too much fragmentation and inertia to change, in addition to power imbalances across all three discussed IC policies. The political fragmentation contributes to the abundance of inconsistent pilots emerging at both central and decentralised levels and to poor policy implementation, adaptation, and evaluation. All of this stands in the way of change towards more coordinated action.

The increasing burden of chronic diseases is a public health problem worldwide, including in developed countries.^1^ Across 27 member countries of the Organisation for Economic Co-operation and Development (OECD), almost one-third of people aged 15 years and over reported living with two or more chronic conditions, on average.^2-5^ In Belgium, the country under study, chronic diseases are responsible for at least 90% of the societal burden of disease including disability and substantial mortality.^6^ Not only in developing countries, but in wealthy countries as well, health systems are struggling to cope with the overstretch related to chronic diseases and responding to associated patient needs as chronic care is organisationally complex.^7-10^ It requires long-term (often lifelong) and coordinated action from different health, social, and policy actors.^7^

 Internationally, these challenges have spawned a policy movement towards ‘integrated care’ (IC).^11,12^ IC can be defined as ‘health services that are managed and delivered in a way that ensures people receive a continuum of health promotion, disease prevention, diagnosis, treatment, disease management, rehabilitation, and palliative care services, at the different levels and sites of care within the health system, and according to their needs throughout their life course.’^13^ Constructs commonly linked to IC include patient-centred care, care coordination, continuity of care, chronic disease management, and integrated healthcare delivery.^13-27^ IC not only leads to better coordination, efficiency, and cost control of care but also improves the quality of care and patient outcomes.^7,28,29^ The contemporary mission of IC policy is thus to improve health system performance across the Quadruple Aim.^30-35^ This entails: (1) improving the quality, safety, and experience of care [individual/patient level], (2) improving population health with a focus on access, equity, the vulnerable and chronically ill [population level], (3) reducing costs of care, whilst creating efficiency and best value for public health system resources [system level], and (4) health worker job satisfaction [individual/caregiver level].^30-36^ However, to achieve this, effective health and social policies are needed to support health systems and facilitate the paradigm shift from curative, episodic, hospital-based, and provider-driven care to a more comprehensive, patient-centred, long-term care approach emphasising the integration of health services.^7,37-42^

 Stakeholders play a key role in the policy process and can influence IC at different stages of the policy cycle: agenda-setting, policy formulation, adoption, implementation, and evaluation.^43^ They can be defined as ‘actors (persons or organisations) with a vested interest (or stake) that are likely to influence decision-making and/or implementation of a policy.’^44^ This definition highlights the role they play in the complex policy process as well as their power and interest in it. Implementation of IC policy is often challenging due to the need for intersectoral, multi-stakeholder action on IC and engagement across different levels and departments of government.^7,22,38,45^ Nevertheless, to enable effective and sustainable policy implementation, public health approaches need to take key stakeholder views into account.^38^ Considering these reasons, stakeholder analysis is a worthwhile tool to understand stakeholders’ perceptions and influence over IC policy.^46^

 In Belgium, a federal state with one federal and five federated governments,^47^ a heterogeneous mix of stakeholders operate and negotiate according to the long tradition of social dialogue,^48^ concertation, and consensus-building as part of its political culture.^49^ Such political context reflects dense, intricate relationships and interactive processes required to develop, adopt and implement IC policies.^50^ This makes Belgium, with its six governments and nine health ministers, a good case for analyses of multi-level collaborative governance and policy coherence across multiple layers of government.^47,51,52^

###  Aim of This Paper

 Since most studies in Health Policy and Systems Research focus on clinical or organisational strategies, macro-level strategies to improve IC remain underreported.^53-55^ Additionally, IC policy implementation has so far been studied with little emphasis on the ‘political determinants of health.’^56^ This paper addresses this gap. A political lens allows the incorporation of *policy and political processes* and* stakeholder power and interest* into the analysis.^39^

 This paper examines how three IC policies in Belgium were developed over the last decade and how stakeholders have played a role in these policies. To describe the IC policy processes and role of stakeholders, three policies were purposely selected: (*a*) the 2009 Type 2 Diabetes (T2D) Care Trajectory,^57^ (*b*) the 2015 Joint Plan on Integrated Care for Chronic Patients,^30^ and (*c*) the 2017 Primary Care Reform in Flanders.^35^ Specifically, this study has two objectives:

1. To examine the policy cycles of three purposefully selected IC policies as well as related IC policies at international, Belgian (federal), and Flemish (federated) levels from 2007 until 2020 via an IC policy timeline, and
2. To uncover the positions, interest, and influence of Belgian (federal) and Flemish (federated) stakeholders on these three policies in different phases of the policy cycle.

Due to the vast amount of actors in Belgium and differences in language, culture, and existing context-specific challenges in Brussels and Wallonia, this study focuses on federal and federated IC policy reforms, as adopted in the region of Flanders, highlighting the complex interactions (including power dynamics) between the federal and Flemish authorities.

###  The Belgian Context

 In Belgium, both federal and federated authorities are responsible for health policy.^49^ Federal authorities are responsible for: (1) the general legislative framework of the health system, (2) regulation of compulsory health insurance, (3) ambulatory care budgets, (4) hospital budgets and programming standards, (5) pharmaceuticals and their price controls, and (6) health professions.^36,49^ Federated entities (Regions and Communities) are responsible for: (1) health promotion and prevention, (2) organisation of primary care and palliative care, (3) maternal and child healthcare, (4) social services and community care, (5) financing hospital infrastructure and medical equipment, and (6) establishing hospital licensing standards.^36^

 Gerkens and Merkur describe how the devolved, federated structure of regions and communities developed through successive rounds of reforms to Belgium’s Constitution carried out between 1970 and 1990.^49^ The most recent, the sixth state reform (2014), led to further decentralisation of public health and healthcare competences.^49,58,59^ The devolution process of public health policy has resulted in a shift in responsibilities from federal to federated levels and led to a competency split between curative care and prevention. Inter-Ministerial conferences have been regularly organised to facilitate collaboration between these levels, currently convening nine health ministers from the federal and all federated entities.^36,49,51^

 Belgium has a social health insurance system, which financially depends on contributions from employers and employees, social insurance, taxes, and out-of-pocket expenditures.^36,49^ The organisation of health services in Belgium is characterised by a large freedom of choice for patients and providers, and remuneration is mainly based on fee-for-service payments.^36,49^ General practitioners (GPs) do not play a gatekeeping role and patients are free to consult any GP or specialist. The 2019 OECD report on Belgium’s health profile highlights *“the health system overall performs well in [...]acute care [...], but many aspects of broader public health and prevention policies could be strengthened to improve health and reduce health inequalities. The main challenges are to strengthen further primary care and to promote greater care coordination for the growing number of people with chronic diseases.”*^36^

 Belgian healthcare organisation and policies are highly influenced by non-governmental stakeholders, including various health professionals’ associations and five private, not-for-profit national associations of sickness funds implementing the national health insurance.^36,49^ Together with the sickness funds, these health professionals’ associations (also known as the syndicates of medical professions) influence healthcare policy by traditional lobbying and representation in advisory bodies, and they are directly involved in executive councils or committees in the National Institute of Health and Disability Insurance (NIHDI).^49^ Patients’ associations have also increasingly been present and lobbying on the policy scene.^49,60^

###  Description of the Three Policy Cases

 In the last decade, a variety of policy initiatives on IC have been developed. Three policies considered key in the evolution of IC in Belgium were selected based on the first round of exploratory document review. This study is a part of the ‘*SCale -Up diaBetes and hYpertension care*’ (SCUBY) project^61^ which seeks to evaluate the implementation of IC for T2D and hypertension. Therefore, these two conditions were the starting point for exploring relevant policies in the field of IC for chronic diseases. Furthermore, the three policy cases were purposefully selected due to their variety in maturity (stage in the policy cycle), approach (disease-specific vs. multi-morbidity), and policy levels (federal vs. federated).

####  Policy (a) Type 2 Diabetes (T2D) Care Trajectory

 In 2009, care trajectories were installed across Belgium for the treatment and follow-up of diabetes as a way to improve quality of care.^41,57^ This involved increasing the focus on IC, linking hospitals with primary care levels, and enhancing collaboration between the patient, GPs, specialists, and other health professionals.^49^ The policy’s intent, as a chronic disease management programme, is consistent with the first goal of the Quadruple Aim. The care trajectory manifested via a contract between these three parties, through which financial incentives for regular consultations, health education, and self-management were given to each of them.^49^ Additionally, local multidisciplinary networks (LMNs) were set up in 2010 as a service to support implementation.^49^ The focus on a single morbidity (diabetes) sets the 2009 T2D Care Trajectory apart from the other two more contemporary policy initiatives that adopt a broader focus on a multi-morbid society from an intersectoral chronic care perspective. This policy has gone through several evaluation cycles and has been adapted over time.

####  Policy (b) Joint Plan and Pilot Projects on Integrated Care 

 In 2015, a National Plan named ‘Integrated Care for Better Health,’ incorporating 18 components of IC,^30^ was approved by all competent federal and federated ministers of public health.^36^ Its vision is based on the ‘Triple Aim,’ and complementary principles of improving equity and job satisfaction for the health professionals.^31,62^

 The policy plan embraced a strong bottom-up strategy through the implementation of 12 regional pilot projects, officially launched in 2018.^36^ The pilot projects aimed to experiment with and test new care and financing models for chronically ill patients within a certain region.^30,32,58^ The plan foresaw juridical freedom or space for actions outside the legal framework, eg, allowing for task shifting. Pilots had to establish a joint governance structure and foresee joint financial management of the project resources.^63^ Via the financial impact of activities of the pilot project, efficiency gains could be made, which could be reinvested in the pilot’s activities. A research consortium, called FAITH.be, was made responsible for the support (scientific guidance) and evaluation of these pilot projects.^64^ This policy, or rather, the pilot projects are in the implementation phase.

####  Policy (c) Primary Care Reform in Flanders

 Unlike the other two, this is a federated (regional) policy. In 2017, the Flanders region endorsed a reform of its primary care system, aiming to achieve more person-centred IC, also based on a participatory and bottom-up approach (at least in the policy formulation stage) and the ‘Quadruple Aim.’^31,35,36^ Its transition programme is extensive, entailing several projects such as the development of new structures, and aims to integrate (the currently fragmented) health and social care.^59^ A central part of the Primary Care Reform was the creation of 60 Primary Care Zones (59 in Flanders and 1 in the Brussels Capital Region).^65^ These were set up in 2019 at a local level to support better coordination, intersectoral collaboration, and improve planning for larger groups of the population.^59^ Each zone is coordinated by a Care Board (or a Care Council), which contributes to integrating care at the local level and strengthening collaboration and coordination between local authorities, primary care professionals, social welfare organisations, associations of people needing care and support, and associations of informal carers and volunteers.^35,59^ This Primary Care Reform aims to be innovative by equal representation of four ‘clusters:’ local authorities, healthcare, social welfare services, and representatives of people needing care and support. Also as part of this reform, the Flemish Institute of Primary Care^66^ was founded to provide expertise and support to the Primary Care Zones, while regional care platforms were set up to enhance cooperation among hospitals and specialised care, centres for dementia, palliative care, and prevention, and mental health networks.^59^ At the time of this study (2019–2020), the Primary Care Zones were in the adoption phase.

Supplementary file 1 shows key stakeholders in these three different policies, visualising and giving insight into the political structure and governing bodies.

## Methods

###  Study Design

 We used a holistic, explorative case study design, which allowed for a comprehensive analysis in line with the paper’s two objectives. As a holistic (single unit) analysis, this case study on IC policy as enacted in the Flanders region embeds the three policy cases described above as sub-units of analysis^67^ These three cases are at different stages of the policy cycle, and allow examining stakeholder interactions and perceptions at different policy phases and their shifting positions throughout the policy process. The first policy offers insight into (over) ten years of policy change (2007–2020), while the latter two more contemporary cases, currently in the stages of implementation and adoption respectively, enable direct observation of policy actors’ perceptions and ongoing interactions during decision-making processes. This provided rich data for the sub-unit analysis (2019–2020).

 We triangulated evidence from literature review and stakeholder interviews to gain insight into the context of care provision in Belgium, whilst exploring the policy processes and intrinsic attributes (position, interest, and power) of stakeholders.

###  Study Population

 Potential interviewees were people considered to be stakeholders in the policy cycle of one or more of the policies described. They were identified and selected via a three-pronged approach^44^: (1) literature review, (2) networking, and (3) snowball sampling. Respectively, this included compiling and reviewing existing information (any publicly available written documents related to the selected policies, including grey literature, policy briefs, and reports); consulting experts who provided further input and feedback; and requesting interviewees to identify key persons playing an instrumental role in the discussed policies. These approaches generated a list of roughly 50 stakeholders with a stake in IC policy, from which 26 were purposively selected for interviews. Stakeholders with higher stakes (interest) and power (influence) were prioritised. High-level officials, such as directors or secretary-generals within organisations, were targeted, but not always available. Consequently, the interviewees were a mix of high-level officials (eg, cabinet staff) and technical staff (eg, from the administration), which included policy-makers, civil servants (Ministry of Health and health insurance), representatives of health professionals’ associations, academics, and patient organisations. Whereas individuals were anonymised, Supplementary file 2 portrays the list of the participants’ organisations, while Supplementary file 3 gives an overview of the full organisation names and their Dutch abbreviations.

###  Data Collection

 During the data collection phase, parallel to the stakeholder identification and selection, *literature review* was conducted, consisting out of an appraisal of grey literature and policy documents. Grey literature review of secondary data—including official (organisation) websites and newspapers—and policy document review were concurrently performed, with the aim to map IC policies at international, national, and Flemish levels and identify policy actors’ roles, actions, and interactions in IC policy processes. This extensive review facilitated the development of an *initial timeline on IC policies* (Supplementary file 4), which was used in the interviews as a starting point for the discussion on IC policies and stakeholders’ positions and interests.

 Following the stakeholder selection and development of the initial IC policy timeline, twenty-six qualitative in-depth* stakeholder interviews*, on the subject of IC and the three policies, were conducted (in Dutch) by the two first authors between April and September 2019, with a semi-structured interview guide (Supplementary file 5). The interviews aimed to gather stakeholders’ views on IC policy implementation in Belgium, in particular, the three policy cases and their roles in it. Written informed consent was offered before participation. One stakeholder requested to retract their data after the interview. Therefore, data from twenty-five interviews were included and analysed. Interviews were audio-recorded and transcribed verbatim by a transcriptionist.

###  Methods for Analysis

*Processual analysis* was used to explore the dynamic policy and political processes (events, actions, and activities) unfolding over time in relation to the three policy cases.^68,69^ The processual analysis was a result of triangulation of both the document review and interviews. Through iterative data collection and analysis—most notably the input from interview participants on the initial IC policy timeline and further review—the initial policy timeline was further refined. The policy timeline thus enabled tracking of the policy cycles of the three selected IC policies as well as other relevant IC policy developments.

*Stakeholder analysis* was used as an approach to ‘generate knowledge about actors to understand their behaviour, intentions, inter-relations, and interests; and to assess the influence and resources they bring to bear on decision-making or implementation processes.’^70^ It entailed a detailed mapping of stakeholders’ attributes, including power, leadership, resources, position, and interest. Data analysis tools were developed based on Schmeer’s guide on stakeholder analysis.^44^ The stakeholder analysis led to three distinct power × position maps, one for each policy case-study. The meaning and way of scoring the stakeholders’ attributes are shown in Table 1.

**Table 1:** Stakeholders’ Attributes, Definitions, and Rating

**Stakeholder Attribute**	**Definition**	**Rating**
Position	Whether the stakeholder supports, opposes, or is neutral about the policy, which is key to establishing whether or not he or she will block the policy implementation.^44^	S (highly supportive) MS (moderately supportive) N (neutral) MO (moderately opposed) O (highly opposed)
Interest	The stakeholder’s interest in the policy, or the advantages and disadvantages that implementation of the policy may bring to the stakeholder or his or her organization^44^; hence the way the stakeholder is affected by the policy.	High Medium Low
Resources	The quantity of resources available to the stakeholder and his or her ability to mobilize them,^44^ used to quantify power. Fischer and Strandberg-Larsen found that this way power can be assessed based on three major factors: financial incentives, technical expertise, and influential (political) position.^71^	1R = technical 2R = political or financial and technical 3R = financial, political, and technical
Leadership	The willingness to initiate, convoke, or lead an action for or against the health reform policy.^44^	+L (leadership) −L (no leadership)
Power	Meanings of power are diverse.^72^ Power is here used as a meta-term: power as resources (R), and power as influence to invoke action, ie, leadership (L).^44,71^ In sum, this entails the ability of the stakeholder to affect the implementation of the health reform policy, which determines the level of force with which the stakeholder might support or oppose the policy.^44^	6-point scale: 6) 3R+L (full power) 5) 3R−L (high power) 4) 2R+L (medium-high power) 3) 2R−L (medium-low power) 2) 1R+L (soft power) 1) 1R−L (little to no power)

 The rating process contained several steps to strengthen the rationale behind a given score. First, the rating was derived from participants’ own views, but also their perceptions of other stakeholders. Second, additional evidence was collected via desk research to back up the scoring if stakeholders’ perceptions on certain attributes were unclear or vague. Third, stakeholders’ attributes were rated independently by the two first authors. Finally, the independent scores and summarised evidence were reviewed by a group of researchers, and consensus scores on stakeholders’ attributes were reached during a research team workshop. At the end of this interpretative rating process, the research group discussed inter-rater reliability (consistency) and internal validity (credibility). The mapping exercise was supported by a stakeholder table (Supplementary file 6), in which these key characteristics were rated for all interview participants. Stakeholders with low power and medium or low interest were omitted from the mapping.

 Nvivo 12.5.0 software was used to analyse the audio transcripts of the interviews through rigorous thematic analysis on the stakeholder attributes (ie, stakeholder analysis). Quotes were derived to support the processual analysis (statements on the IC policy cycle) and stakeholder analysis (statements clarifying a stakeholder’s position or interest). The flowchart in Supplementary file 7 displays the process from data collection to analysis and the use of data sources.

## Results

###  Processual Analysis: A Timeline of IC Policy and Political Processes

 The exploration of the IC policy cycles led to an intricate mapping of key policy documents and events, as shown in Figure. The three key policies under analysis are in bold.

**Figure:**
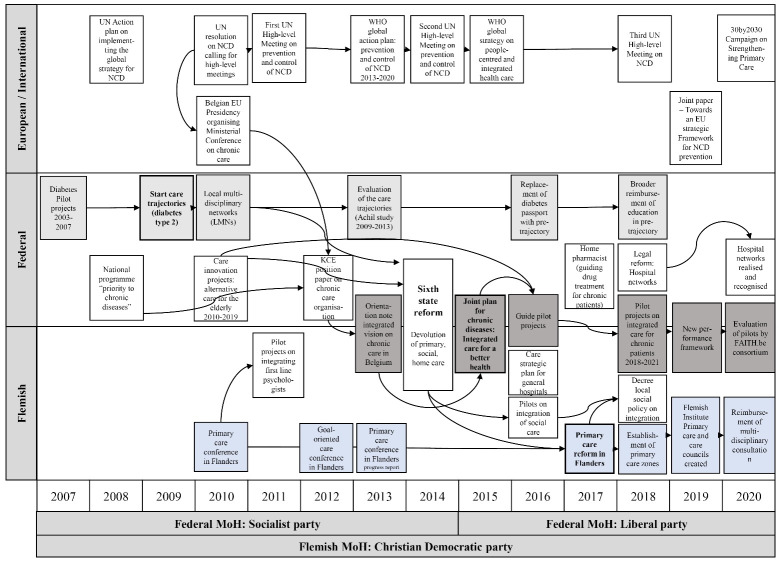
Timeline Portraying Policy Processes on IC.^4,5,11,14,30,35,60,73-90^ (1) Blue indicating initiatives and events on the federated (Flemish) level; (2) Light grey indicating initiatives related to the diabetes care trajectory; (3) Darker grey indicating all initiatives leading up to the launch of the pilot projects on IC. Abbreviations: IC, Integrated Care; EU, European Union; WHO, World Health Organization; UN, United Nations; NCD, non-communicable disease; MoH, Ministry of Health; KCE, Federal Knowledge Centre for healthcare.

 Figure shows a web of policies on three levels, including European/international, federal, and federated (Flemish) levels. Upon further examination of the linkages, these policy documents and strategic plans very often arise from or produce pilot projects, both on federal and federated levels. The broad variety of pilot projects in Belgium (including diabetes pilot projects, projects concerning alternative care models for vulnerable elderly, the integration of primary care psychologists, integrated social reception, and IC for chronic patients) supports the belief of several interview participants that much is ‘being done’ in Belgium’s fragmented field of care integration, yet insufficient time is spent on evaluation:

*“Everyone talks about projects, but in the long run, you don’t know what the difference between one and the other is (...) Integrated care policy is not so great in Belgium. All pilots, everything is a pilot” *(ID19).

 Nevertheless, these many attempts and initiatives have given attention to IC. Many stakeholders voiced their belief in IC and the need for a paradigm shift from the currently strong supply-oriented (provider-driven) system to a needs-oriented (patient-centred) system. Some doubted whether this same belief in IC and desire for change was reflected in the field. One stakeholder spoke about growing awareness in the field of practice yet hinted at the problem of a policy-implementation gap:

*“In the field [in practices], you can see growing awareness [on IC]. But naturally not everybody. But the awareness is there, like we have to collaborate and we want to participate via different possible actions and structures. However, if you are looking from the perspective of the person, I think too much is not yet aligned and much remains to be done. From all the things in the policy vision papers and the national plan on chronic diseases, only little pieces have been enforced” *(ID11).

 Amid these policy developments, the sixth state reform has been a catalyst for political change but it was one of the most commonly named barriers to IC. The processual analysis highlights differences in political agendas and underlying initiatives at federal and Flemish levels which can be related to the absence of a shared vision and the fragmentation of power following the sixth state reform. Its broad implications point to the highly political nature of healthcare and are associated with concerns regarding accountability, complexity, transparency, and leadership:

*“The sixth state reform is a disaster; it has increased fragmentation, it has increased the tension between those who take the lead. So there is no mutual loyalty [between levels/policy makers]” *(ID23).

 Ultimately, this fragmentation of responsibilities among federated and federal levels impedes care integration as different aspects of the care continuum are led by different governments, offering fundamental challenges for policy coherence and decision-making.

###  Stakeholder Analysis: Policy-Specific Power × Position Maps

 Three position maps are presented in Tables 2-4. The attributes of power (consisting of resources and leadership) and position were analysed for each policy.

**Table 2:** Position Map on the T2D Care Trajectory (2009)

Position * Power	**High Support** **++**	**Moderate Support** **+**	**Neutral** **+/−**	**Moderate Opposition** **−**	**High Opposition** **−−**
Full power					
High power				Federal cabinet, Flemish cabinet	
High-medium power	Medical syndicate 2				
Low-medium power	NIHDI, medical syndicates 1+3	Sickness funds		Flemish administration	
Soft power	Diabetes nurses, diabetes association				
No power		Dieticians, self-employed nurses, salaried nurses			

Abbreviations: NIHDI, National Institute of Health and Disability Insurance; T2D, type 2 diabetes.

**Table 3:** Position Map on the Joint Plan and Pilot Projects on IC (2015 & 2018)

Position * Power	**High Support** **++**	**Moderate Support** **+**	**Neutral** **+/−**	**Moderate Opposition** **−**	**High Opposition** **−−**
Full power					
High power				Flemish cabinet	Federal cabinet
High-medium power					
Low-medium power	Federal administration, NIHDI, FAITH.be	Sickness funds, medical syndicate 2, care network, Flemish patients	Flemish administration	Medical syndicate 1, salaried nurses	Medical syndicate 3
Soft power		Pharmacists			
No power				Self-employed nurses	

Abbreviations: IC, Integrated Care, NIHDI, National Institute of Health and Disability Insurance.

**Table 4:** Position map on the Flanders’ Primary Care Reform (2017)

Position * Power	**High Support** **++**	**Moderate Support** **+**	**Neutral** **+/−**	**Moderate Opposition** **−**	**High Opposition** **−−**
Full power	Flemish cabinet				
High power	Flemish administration				
High-medium power	Medical syndicate 1, Flemish patients	Salaried nurses			
Low-medium power	Flemish cities-municipalities, sickness fund 1, home nurses	Care network	Federal cabinet		
Soft power	Pharmacists, dieticians				Medical syndicate 3
No power		Medical syndicate 2		Self-employed nurses	

####  (a) Stakeholder Support and Power Post-implementation

Table 2 shows stakeholders’ power dynamics and position on the 2009 T2D Care Trajectory, post-implementation. Extreme positions have likely stagnated over time as acceptance and normalisation set in. Historically, support for care trajectories has been highest among physicians’ associations and syndicates who helped conceptualise them. One stakeholder mentioned that interest groups and particularly specialists still hold on to this policy.

 According to interviewees, the success factors of the T2D Care Trajectory are its connection to, and the employment of, LMNs and incentives for the physicians. Other than chronic kidney disease, it has never been extended to other conditions due to budgetary constraints and new insights that support a multi-morbidity approach. Yet, many stakeholders supported or moderately supported it. Dieticians and nurses who moderately supported it mentioned the insufficient reimbursement for their role as health educators (with a salary disproportionate to their workload). This is an illustration of the power struggle between health professionals, pointing at budgetary corporatism. Moderate support could further be explained by the largely positive, yet mixed feelings of interviewees. They considered the T2D Care Trajectory a good first attempt of IC and innovative at the time, but almost unanimously agreed that full care integration could not be reached through this disease-specific programme. One stakeholder called it a “*modest form of a disease management programme.*” Other stakeholders viewed its added value in the clear task delegation, professionalization, and knowledge sharing. Some stakeholders pointed out that the evaluation was substandard:

*“If more evaluation and adaptation had been done, then we would have had more progress in these last ten years than we have today” *(ID2).

 In recent years, financial and political support has been declining, not just for the pre-trajectory at the federal level, but also for the coordinating and supporting structure—the LMNs—at the Flemish level, where they have been transferred to since the state reform, and where they were ‘absorbed’ by the Primary Care Zones. The Flemish cabinet, its administration, and federal cabinet all had moderately opposed positions towards continued policy implementation, due to their lack of financial support and interest. Politics thus had a strong influence on the continuation of this policy. Additionally, the state reform of 2014 and the fragmented governance structure of Belgium, have had repercussions on the T2D Care Trajectory. One stakeholder explained the discontinuation of the LMNs and the complexity due to fragmented competencies:

*“I had to say to the LMNs, when they were still federal, ‘you must not do prevention, detection, screening, large-scale prevention, because that is the responsibility of the federated states.’ Now the LMNs are with the federated states and are no longer allowed to work with the NIHDI, because it is the competence of the federated states. I find that personally, on an operational level, I find that difficult and not logical” *(ID25).

 Although the T2D Care Trajectory in Flanders has been effective, many consider it somewhat outdated due to its solely medical and pathology-specific approach to IC.

####  (b) Stakeholder Support and Power During Implementation

Table 3 shows the position map of the Joint Plan on IC, or more specifically, its implementation via the IC pilot projects since 2018. Stakeholders pointed out two crucial features of the overall plan, namely its *ambitious* and *experimental* nature. Stakeholders often related the *ambitious* character of this policy to the visionary (triple) aim of the policy and the inclusion of all levels of government in the negotiations and agreements. They also noted how other local, regional, and federal actors were involved through the bottom-up development of this policy plan. Sickness funds and local authorities, however, were not named as mandatory partners in the policy plan, yet turned out to be important partners in the pilot implementation. Furthermore, many interviewees saw the number of pilot projects as highly ambitious and the implementation time too short. The most supportive interviewees viewed *experimenting* with the care and financing model as key to the trial-and-error process, and recognised that there is not one success formula (but rather multiple best practices or pathways). Notwithstanding the initial experimental set-up, implementation was restricted because of two developments. First, the federal government decided to install a performance framework that introduced new rules to calculate efficiency gains and adopted a more controlling approach, favouring short-term successes over longer-term efficiency gains. Second, the royal decree on the Joint Plan on IC was ruled unconstitutional, as the foreseen redistribution of responsibilities between federal and federated levels was incongruent with the law on budget transfers between governments.^91^ As a result, reinvesting efficiency gains became near impossible since pilot projects could not finance activities that were the responsibility of regional (federated) entities, eg, substitute tasks from nurses to home care workers. This legal and political conundrum had huge repercussions on their implementation and success.

 The dissolution of the legal basis of the policy led to the regional/federated and federal authorities being disunited in their (governance) approach to IC. Pilot projects became federal rather than a joint project between federal and federated governments. This resulted in changes in the administrative obligations for pilot projects. Whereas people implementing the pilot projects expected that federal and federated entities would govern in concertation and co-decide, they were faced with obstacles from both sides. At the federated level, governments barely shared information on their plans and were often absent during policy meetings.

*“We always invite federated states. Only Wallonia comes, the others don’t. My sense is that they just don’t care, they’re busy with them [their own things] and other things do not interest them” (ID17). *

*“The intention was also to inform each other because the information is always in one direction, from federal to federated state, I am not only talking about Flanders, but never in the other direction. So Flanders is busy with everything and we don’t know that. Unless we’re really fishing for it, ask about it. So integrated politics. That is important because the projects experience this almost every day, that they notice that it does not click there (…)” *(ID17).

 Despite their claim of supporting pilot projects, we observed two reasons for Flanders’ diminished support (towards ‘a moderate opponent’): (1) the prohibition on transferring budgets made the Flemish authorities less resolved to help out pilot projects, and (2) the Flemish cabinet’s focus shifted to developing its own Primary Care Reform.

 At the federal level, multiple issues appeared. Many interviewees associated the retirement of the previous director of NIHDI, originator of the plan, with the loss of leadership and lack of direction on IC. The 2014 elections resulted in a change of political vision and shift in priorities. Some stakeholders considered the follow-up by the federal cabinet on the Joint Plan on IC restraining, as politicians actively wanted to scale down the policy and blocked its full implementation. One stakeholder claimed: *“I think [the federal head of cabinet] devours a pilot project daily” *(ID19).

 The only interviewees expressing full support for the projects were the respondents from the Federal Public Service, NIHDI, and FAITH.be. They felt frustrated about the lack of political support, as the federal cabinet—the most resourceful stakeholder—disregarded the pilots and became one of its strongest opposers, given its political and financial power. One stakeholder argued that trust dissipated due to the lack of clarity within this politically charged legal quarrel. It is highly likely that with the drop in political support and dissolved legal foundation, stakeholders’ support and the originally reported enthusiasm of partners in the projects declined. Two interviewees, one from the medical syndicate 1 and a salaried nursing association, explicitly rated the implementation a disaster, because of the lack of political support and leadership and due to the juridical and financial tangle. Medical syndicate 3 was pointedly against any reform towards IC, whereby health professionals are bound to register patients’ data and confer more, moving the emphasis away from care delivery to interprofessional consultation and administrative work. Such changes require time investment, which especially self-employed practitioners consider heavy and voluntary.

 Barriers to this second policy are rooted in Belgium’s multi-level fragmented governance structure. Stakeholders agreed on this part:* “There is no integrated politics;” “The power is missing;” “Because of the sixth state reform, integrated care was made impossible;” “Integration of governments did not succeed.”*

####  (c) Stakeholder Support and Power During the Policy Adoption Phase

Table 4 shows the stakeholders’ power and positions regarding the 2017 Flanders’ Primary Care Reform in the adoption phase. Many actors were involved in this reform and its development since the start of the 2010 Primary Care Conference. This ensured motivation and common ground among the partners. Because of the political continuation of the Flemish government over ten years—across two legislatures in which the same party delivered the Ministry of Health—the reform could come to fruition, with investment in the development and adoption of the reform.

 Nearly all interviewed stakeholders supported this reform, except for two players from the self-employed medical sector. First, the more conservative medical syndicate 3 (specialists and GPs), has consistently voiced its stance against IC and more interprofessional meetings. They also were unhappy with the exclusion of secondary care specialists as partners in the reform and not being given a seat within the Institute of Primary Care. Second, self-employed nurses were moderately opposed given their voluntary (unpaid) time investment and subsequent limited involvement in the Care Councils of the Primary Care Zones. Several stakeholders from the private sector voiced concern that they, as self-employed medical professionals, may be disadvantaged. They argued that salaried social workers have more time for negotiation and concertation at such governing structures.

 Key initial successes considered by the interview participants were: the appointment of patients and local authorities as mandatory partners in the governance of the Zones; the integration of the medical and social sector; and the systemic approach taken through the set-up of geographical structures/zones.

 Of financial stakeholders, only sickness fund 1 was seen as a strong supporter on this map (sickness fund 2 had low interest and power in this reform). The reason behind this is that sickness fund 1 leans closer to the ‘political ideology’ of the Flemish minister of health leading the reform, and therefore has more influence and higher interest.

 There also were a few critical voices. One particular stakeholder raised two points of criticism: the financial support to the projects of the Primary Care Reform remains unclear; and although a network structure for collaboration has been set up, its purpose remains unclear:

*“What is still unclear is where it should lead? So in the Primary Care Decree, the main message is still cooperation between existing ones, yes? The message is not yet that strong on change. Yes? It is not by putting actors together that you get a new project as a result. Collaboration is important, so working together around a patient is certainly good. Coordination is certainly good, but it is not yet integration. Yes? (...)” *(ID23).

 Challenges to the ongoing implementation of the reform remain. Without clear policy objectives, the reform’s success depends on the mentality and change management in the field. A few stakeholders expressed fear of the Care Councils becoming discussion hubs instead of decision-making structures focused on action. Generating visible and substantive change is a challenge.

## Discussion

 This study examined the policy process and the influence of stakeholders on three IC policies of the last decade in Belgium. Our findings show that most interview participants believed in the need for change towards a more patient-centred system, but also that this intention does not stroke with the current strongly provider-driven system and institutional design in Belgium. Furthermore, the results illustrate that Belgium’s political structure is characterised by fragmentation, inertia to change, and power imbalances affecting all pivotal policies for IC. This constrains a change process towards more coordinated implementation.

###  The Impact of Belgium’s Institutional Design on Policy-Making

 The provider orientation is reflected in the strong representation of medical syndicates and their power in policy-making at the federal level. Health professionals remain very influential in insurance committees within the NIHDI at the federal level, while the beneficiary, the patient, is not represented within this organ. The Flemish government has attempted to address this power imbalance and the near absence of the social welfare sector in health reforms, through the representation of patients, the municipality, and the medical and social sector in the Care Councils of Primary Care Zones. Flanders’ way of creating structural, collaborative governance platforms is innovative. Change at the federal level appears slower because of inter-administration coordination, the large number and inertia of co-decisive organs, and the conflicting political priorities by governing parties at different levels. In general, political fragmentation is a widely acknowledged problem resulting from Belgium’s complex institutional design.

###  Political Fragmentation Leads to Lack of Policy Implementation

 Despite and because of the profound political fragmentation, various policy initiatives have been recently developed in Belgium at both central and decentralised levels. The policy processual analysis portrayed on the timeline showed the evolution and entanglement of different policy initiatives at different levels and how it has characterised the Belgian health (and social) sector. A major finding of this processual analysis was the abundance of pilot projects and the lack of follow-up or evaluation.

 The stakeholder analysis exposed tensions between federal and federated government levels in terms of overlapping and fragmented competences. The 2014 state reform introducing a partial power shift from federal to federated levels exacerbated this tension and was repetitively described by stakeholders as a key barrier to IC policy implementation. The political fragmentation contributes to the abundance of inconsistent pilots emerging and to poor policy implementation, adaptation, and evaluation. Further repercussions are that goals for better health system performance written out in the Triple (or Quadruple) Aim, including health equity, remain far out of reach.

###  Healthcare Politics

 In all three policy cases, we observed a lack of financial support for primary care and IC reforms. In this regard, Stuckler et al argue that despite repeated calls to action on chronic diseases over the past decade, there is a critical lack of funds and resources available for chronic disease control, also recognising that ultimately, the failure to prioritise and act on chronic diseases is a political, rather than a technical issue.^92^

 Indeed, Belgian health policy and practice remain determined by medical corporatism,^93^ indicating the large influence interest groups have on the concertation structures within the NIHDI. Both corporatism and concertation are key components of Belgium’s political system.^48,93^ Gaventa notes that in this regard, power remains complex, as its nature and expressions continue to evolve.^72^ The author argues: *“The very spread and adoption by powerful actors of the language and discourse of participation and inclusion confuses boundaries of who has authority and who does not, who should be on the ‘inside’ and who is on the ‘outside’ of decision-making and policymaking arenas.”*^72^ This is clearly true for Belgium, as even among the interviewed stakeholders, there was confusion about where the real power is situated in IC. This becomes harder to define when more actors are involved and power becomes too fragmented.

###  IC Reform Durability?

 Out of the three policy cases, the T2D Care Trajectory in Flanders has been effective but has become outdated. While it has stimulated transmural collaboration, it offers an incomplete approach to IC for two reasons: (1) it is embedded solely in the medical sector, and (2) it creates inequity by disease. These are the partial reasons behind the decline in financial support by the federal and Flemish health ministry.

 The two contemporary IC policies—the Primary Care Reform in the adoption phase and the federal Joint Plan and pilot projects on IC in the implementation phase—highlight a varied degree of reform durability post-enactment. The Flemish Primary Care Reform has known success post-adoption due to stakeholders’ strong supportive positions. The reform offered a strong base—a legal and geographical frame—to improve collaboration between the social and medical sectors and worked on formalising ties with users and local policy. Durability questions are: how will self-employed medical workers connect to salaried social workers? Will the reform facilitate substantive care integration? Will patients, informal caregivers, and municipalities actually obtain more power because of their role in the Care Council in the Primary Care Zones, or will their representation risk becoming tokenistic?

 The durability of the federal pilot projects seems less certain, despite their high potential. The drop in political support, lack of leadership and dissolved legal foundation led to waning stakeholders’ support and heavy criticisms on the structural problems that the pilot projects and their partners encountered.

###  Integration or Fragmentation?

 Our study’s main conclusion is that Belgium’s current fragmented political structure does not stroke with the IC paradigm. Despite all efforts in Belgium towards integration, (political) fragmentation trumps care integration. Not just the interviewed stakeholders, but several other voices across the health sector have been calling for the further decentralisation of healthcare competences on one hand, and the re-federalisation of healthcare on the other, to address the profound political fragmentation.^51,94-99^ This dual pull in opposite directions reflects the political complexity with different political colours ruling at different policy levels and the highly politicised power game that is healthcare in Belgium. Despite this current impasse on centralisation vs. decentralisation, all interview participants recognised the healthcare competency split leading to political inertia as the cause of policy ineffectiveness.

###  Strengths and Limitations

 The strengths of our study relate to the wide range of stakeholders interviewed from different levels and the depth of our analysis using multiple methodologies and theoretical frameworks to look at stakeholders and policies. We had a good mix of participants, including stakeholders in top positions (highest power in their organisation) and those with more technical expertise (political or operational level) concerning IC. The data analysis posed several challenges. Positions regarding a policy initiative were not always clearly stated by everyone. Personal views of individuals and their organisational position cannot easily be distinguished and might lead to bias into the scores early on. Triangulation of sources was used to counter this effect to the degree possible. Our stakeholder selection was limited to the healthcare and social welfare sector; however, views of actors from other sectors that could influence IC policies could have widened the range of barriers and solutions to IC, for instance, economic policies. The main limitation of this research relates to the nature of the stakeholder analysis, which induces interpretive judgements^70^ to rate stakeholder attributes and using static models to represent dynamic stakeholder relationships.^100^ We addressed this constraint in two ways. First, processual analysis was used as a method to showcase the respective policy processes and emergence of policy documents. Second, three policies were chosen in different stages of the policy cycle. This highlighted how positions can change throughout the policy process and are most challenged in the piecemeal implementation stage. It is therefore important to note that these stakeholder maps are subject to constant change. Regarding the processual analysis visualised via a detailed and extensive IC policy timeline, it should be noted that this timeline is not exhaustive, as it aimed to display the evolution of the three policy cases and other related developments within the federated (Flemish), federal (national) and international context within the last decade. Certain policy topics related to IC (elaborations on goal-oriented care,^41,101^ capitation-based practices,^102^ the Global Medical Record,^103^ mental health, cancer, e-health plans, and health promotion such as nutrition) were out of the scope of this exercise. Despite having built on literature review and stakeholder input, the choice for an in-depth case study design made us focus on selected policies which might have led to a selection bias.

###  Implications

 This case study provides an important insight into the power and policy processes towards IC in a fragmented supply-oriented healthcare system. The main implication for further policies and implementation of IC is that political fragmentation needs to be overcome, in one way or another, to make comprehensive progress. The boundaries of incremental changes have been reached, especially in times where new threats (such as coronavirus disease 2019) emerge and resources will be only more restrained. Facilitating IC requires large decisions in the health and adjacent sectors on financing^104^ and on the distribution of power.^42^ Further research on the evolvement of positions, interests, and power will expose these (difficult) choices and the effects of not making them. Not only the analysis of stakeholder attributes but also research on how public health policy competences and reform durability of IC policies evolve in Belgium can shed light on the complex policy processes towards IC and can inform other countries on political pitfalls.

## Acknowledgements

 We thank all interview participants that provided us with data and information for this study. Comments and insights from Prof. Dr. Bart Criel in the stakeholder identification and selection phase and from Dr. Elien Colman in the data collection and analysis process are gratefully appreciated. We also wish to thank the entire FAITH.be team for their facilitation and cooperation. Finally, we wish to thank Ritwik Dahake for his help and collaboration on the editing of this paper.

## Ethical issues

 The Ethics committee of Antwerp University Hospital approved this study (registration number B300201940005).

## Competing interests

 MM reports grants from the NIHDI, grants from European Commission, during the conduct of the study; and Roy Remmen and Sibyl Anthierens coordinated an evaluation project for integrated chronic care projects that was ordered by the Federal Government of Belgium and funded by the NIHDI (https://www.integreo.be/nl/pilootprojecten/evaluatie). As such, these two authors were part of FAITH, which stands for ‘Federated consortium for Appraisal of Integrated care Teams in Health.’

## Authors’ contributions

 MM and KD were responsible for the data collection, processual analysis, analysis of interviews, independent scoring on stakeholder attributes (as part of the stakeholder analysis) and the writing. MM, KD, SVB, EW, RR SA, and JVO participated in the research workshop on consensus scores on stakeholders’ attributes (as part of the stakeholder analysis). MM, KD, SVB, WVD, SA, and JVO were responsible for the research design. All authors contributed to the drafting of the manuscript. All authors have read the manuscript and approved for submission.

## Funding

 This study is part of the ‘SCale-Up diaBetes and hYpertension care’ (SCUBY) project, funded by the European Union and was supported by funding from the Horizon 2020 programme of the European Union [grant number 825432]. Data collection (interviews) and preparation of this study were done in collaboration with the FAITH.be project.

## Supplementary files

Supplementary file 1. Stakeholder Mapping.

Supplementary file 2. List of Participants (Stakeholders’ Organisations).

Supplementary file 3. List of Participants (Full Names of Stakeholders’ Organisations).

Supplementary file 4. Original Timeline.

Supplementary file 5. Interview Guide.

Supplementary file 6. Stakeholder Table With Scores (All Participants).

Supplementary file 7. Flowchart Showcasing Data Collection and Analysis Process.
