# Supplementary material for: Integration or Fragmentation of Health Care? Examining Policies and Politics in a Belgian Case Study
Source: Int J Health Policy Manag. 2021 Jul 6;11(9):1668–81. doi: 10.34172/ijhpm.2021.58 (PMC9808233; doi:10.34172/ijhpm.2021.58)
Supplement: Supplementary file 1 — Stakeholder Mapping. [file ijhpm-11-1668-s001.pdf]

Article title: Integration or Fragmentation of Health Care? Examining Policies and Politics in a Belgian Case Study

Journal name: International Journal of Health Policy and Management (IJHPM)

Authors' information: Monika Martens[1,2]*¶, Katrien Danhieux[2]¶, Sara Van Belle[1], Edwin Wouters[3,4], Wim Van Damme[1], Roy Remmen[2], Sibyl Anthierens[2], Josefien Van Olmen[2]

[1]Department of Public Health, Institute of Tropical Medicine, Antwerp, Belgium.

[2]Department of Family Medicine and Population Health (FAMPOP), Faculty of Medicine and Health Sciences, University of Antwerp, Antwerp, Belgium.

[3]Centre for Population, Family & Health, Department of Social Sciences, University of Antwerp, Antwerp, Belgium.

[4]Centre for Health Systems Research & Development, University of the Free State, Bloemfontein, South Africa.

¶ Both authors contributed equally to this paper.

(*Corresponding author: mmartens@itg.be)

Supplementary file 1. Stakeholder Mapping

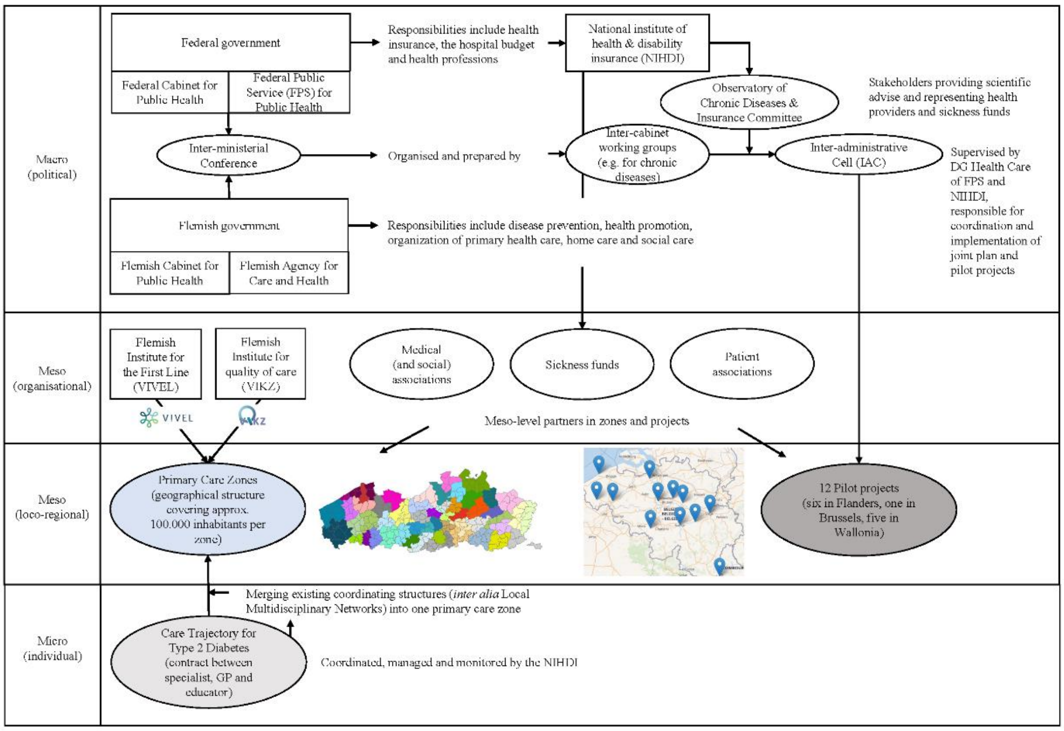

Note:

Stakeholders in three key policy initiatives on integrated care in Belgium: Type 2 Diabetes Care Trajectories (adopted in 2008), National Pilot Projects (adopted in 2018) and the Primary Care Zones (adopted in 2019) in Flanders. The three policies are quite distinct. The care trajectory is a micro-level contract; the pilot projects on IC involve stakeholders from all governmental levels; while Flanders is organising its own reform, building on the same goals, but focused specifically on strengthening primary care. [30,35,36,57,66,73]
