# Supplementary material for: Integration or Fragmentation of Health Care? Examining Policies and Politics in a Belgian Case Study
Source: Int J Health Policy Manag. 2021 Jul 6;11(9):1668–81. doi: 10.34172/ijhpm.2021.58 (PMC9808233; doi:10.34172/ijhpm.2021.58)
Supplement: Supplementary file 2 — List of Participants (Stakeholders’ Organisations). [file ijhpm-11-1668-s002.pdf]

Article title: Integration or Fragmentation of Health Care? Examining Policies and Politics in a Belgian Case Study

Journal name: International Journal of Health Policy and Management (IJHPM)

Authors' information: Monika Martens[1,2]*¶, Katrien Danhieux[2]¶, Sara Van Belle[1], Edwin Wouters[3,4], Wim Van Damme[1], Roy Remmen[2], Sibyl Anthierens[2], Josefien Van Olmen[2]

[1]Department of Public Health, Institute of Tropical Medicine, Antwerp, Belgium.

[2]Department of Family Medicine and Population Health (FAMPOP), Faculty of Medicine and Health Sciences, University of Antwerp, Antwerp, Belgium.

[3]Centre for Population, Family & Health, Department of Social Sciences, University of Antwerp, Antwerp, Belgium.

[4]Centre for Health Systems Research & Development, University of the Free State, Bloemfontein, South Africa.

¶ Both authors contributed equally to this paper.

(*Corresponding author: mmartens@itg.be)

Supplementary file 2. List of Participants (Stakeholders' Organisations)

Stakeholder group and organisation name	Number of participating organisations
**Policy**<br>Federal Cabinet for Public Health (PH)<br>Federal Public Service (FPS) for PH administration<br>Flemish Cabinet for PH<br>Flemish PH administration<br>Flemish cities and municipalities (association)	5
**Finance (National Health insurance)**<br>National Institute of Health and Disability Insurance (NIHDI)<br>Sickness fund 1<br>Sickness fund 2<br>Inter-mutualistic College	4
**Health (and social) care provision**<br>Medical syndicates: 3<br>Nursing associations: 4<br>Pharmacists: 1<br>Flemish dieticians: 1<br>Care network (representing hospitals, elderly care, and mental health care): 1	10
**Scientific**	3
**User**<br>Flemish Patients<br>Diabetes Association	2
**Total number of participating organisations**	**24**
3 separate interviews at NIHDI	+2
**Total number of interviews conducted**	26
1 retracted	-1
**Total number of interviews analysed**	**25**
