# Supplementary material for: Integration or Fragmentation of Health Care? Examining Policies and Politics in a Belgian Case Study
Source: Int J Health Policy Manag. 2021 Jul 6;11(9):1668–81. doi: 10.34172/ijhpm.2021.58 (PMC9808233; doi:10.34172/ijhpm.2021.58)
Supplement: Supplementary file 3 — List of Participants (Full Names of Stakeholders’ Organisations). [file ijhpm-11-1668-s003.pdf]

Article title: Integration or Fragmentation of Health Care? Examining Policies and Politics in a Belgian Case Study

Journal name: International Journal of Health Policy and Management (IJHPM)

Authors' information: Monika Martens[1,2]*¶, Katrien Danhieux[2]¶, Sara Van Belle[1], Edwin Wouters[3,4], Wim Van Damme[1], Roy Remmen[2], Sibyl Anthierens[2], Josefien Van Olmen[2]

[1]Department of Public Health, Institute of Tropical Medicine, Antwerp, Belgium.

[2]Department of Family Medicine and Population Health (FAMPOP), Faculty of Medicine and Health Sciences, University of Antwerp, Antwerp, Belgium.

[3]Centre for Population, Family & Health, Department of Social Sciences, University of Antwerp, Antwerp, Belgium.

[4]Centre for Health Systems Research & Development, University of the Free State, Bloemfontein, South Africa.

¶ Both authors contributed equally to this paper.

(*Corresponding author: mmartens@itg.be)

Supplementary file 3. List of Participants (Full Names of Stakeholders' Organisations)

**A) Regulation/Policy: 5**
Federal level:<br>1. Federal Cabinet for Public Health<br>2. Federal Public Service (FPS) of Health, Food Chain Safety and Environment (Federal administration)
Flemish level:<br>3. Flemish Cabinet of Welfare, Public Health and Family<br>4. Flemish Agency for Care and Health of the Flemish Ministry of Welfare, Public Health and Family (Flemish administration)<br>5. Flemish Association of Cities and Municipalities (VVSG)
**B) Finance/Health Insurance: 4**
6. National institute of health & disability insurance (NIHDI)
7. National Inter-mutualistic College (NIC) ; i.e. joint college of sickness funds
8. Christian sickness fund (Sickness fund 1, CM)
9. Socialist sickness fund (Sickness fund 2, Socmut)
**C) Health (and social) care provision: 10**

10. Medical association of GPs (Medical syndicate 1, Domus Medica)	
11. Medical association of GPs and specialists (Medical syndicate 2, ASGB)	
12. Belgian association of doctor syndicates (Medical syndicate 3, BVAS)	
13. General Pharmaceutical Association (Pharmacists)	
14. Care Network (ICURO) of hospitals, elderly care and mental health care	
15. Flemish association of dieticians (dieticians, VBVD)	
16. Flemish association of Diabetes Nurses (BVVDV)	
17. Flemish association of self-employed nurses (VBZV)	
18. Network of homecare workers and nurses (Zorggezind)	
19. Association of salaried home nurses (WGK)	
**D) Scientific stakeholders: 3**	
20. Federal Knowledge Centre for health care (KCE)	
21. Medical Department at Free University of Brussels	
22. FAITH.be research consortium (support and evaluation integrated care pilot projects)	
**E) User & patient groups: 2**	
23. Flemish Patient Platform	
24. Flemish Diabetes association (Diabetes Liga)	

Note:
(1) Stakeholders are clustered according to their health system function: A) Regulation (policy makers/politicians and public administrators; B) Finance; C) Provision (practitioners, labour & health professionals' associations); D) Science (scientific partners and stakeholders, such as academia/research institutes; E) User (patient groups).
(2) Dutch abbreviations are added between parentheses to specify the stakeholder to Belgian/Flemish audiences.
