# Supplementary material for: Integration or Fragmentation of Health Care? Examining Policies and Politics in a Belgian Case Study
Source: Int J Health Policy Manag. 2021 Jul 6;11(9):1668–81. doi: 10.34172/ijhpm.2021.58 (PMC9808233; doi:10.34172/ijhpm.2021.58)

Article title: Integration or Fragmentation of Health Care? Examining Policies and Politics in a Belgian Case Study

Journal name: International Journal of Health Policy and Management (IJHPM)

Authors' information: Monika Martens[1,2]*¶, Katrien Danhieux[2]¶, Sara Van Belle[1], Edwin Wouters[3,4], Wim Van Damme[1], Roy Remmen[2], Sibyl Anthierens[2], Josefien Van Olmen[2]

[1]Department of Public Health, Institute of Tropical Medicine, Antwerp, Belgium.

[2]Department of Family Medicine and Population Health (FAMPOP), Faculty of Medicine and Health Sciences, University of Antwerp, Antwerp, Belgium.

[3]Centre for Population, Family & Health, Department of Social Sciences, University of Antwerp, Antwerp, Belgium.

[4]Centre for Health Systems Research & Development, University of the Free State, Bloemfontein, South Africa.

¶ Both authors contributed equally to this paper.

(*Corresponding author: mmartens@itg.be)

Supplementary file 4. Original Timeline

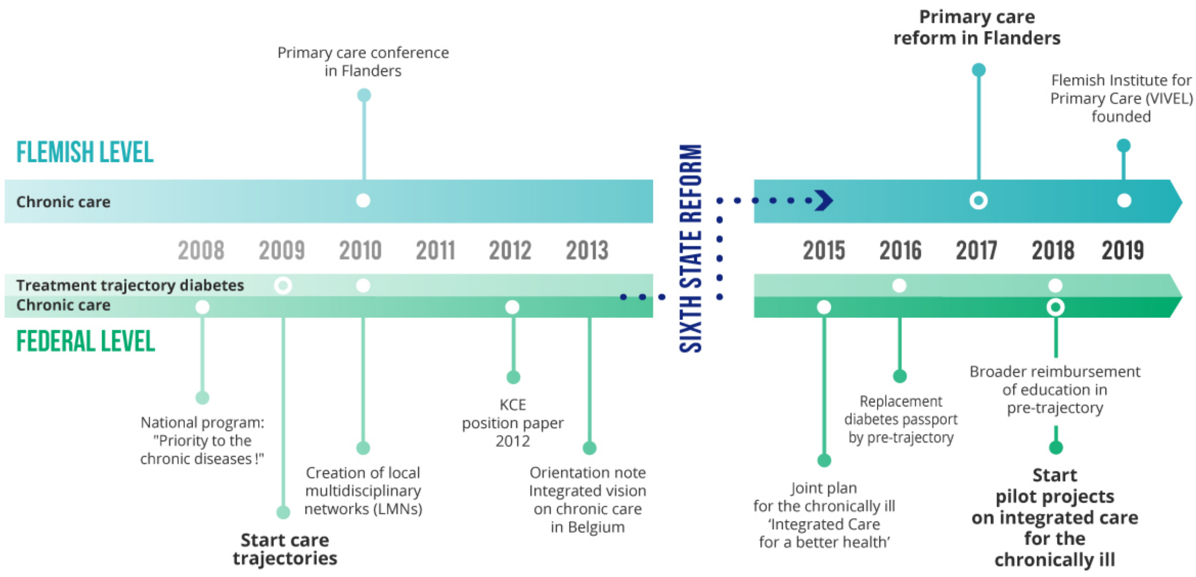

Supplement: Supplementary file 4 — Original Timeline. [file ijhpm-11-1668-s004.pdf]
