# Supplementary material for: Integration or Fragmentation of Health Care? Examining Policies and Politics in a Belgian Case Study
Source: Int J Health Policy Manag. 2021 Jul 6;11(9):1668–81. doi: 10.34172/ijhpm.2021.58 (PMC9808233; doi:10.34172/ijhpm.2021.58)
Supplement: Supplementary file 5 — Interview Guide. [file ijhpm-11-1668-s005.pdf]

Article title: Integration or Fragmentation of Health Care? Examining Policies and Politics in a Belgian Case Study

Journal name: International Journal of Health Policy and Management (IJHPM)

Authors' information: Monika Martens[1,2]*¶, Katrien Danhieux[2]¶, Sara Van Belle[1], Edwin Wouters[3,4], Wim Van Damme[1], Roy Remmen[2], Sibyl Anthierens[2], Josefien Van Olmen[2]

[1]Department of Public Health, Institute of Tropical Medicine, Antwerp, Belgium.

[2]Department of Family Medicine and Population Health (FAMPOP), Faculty of Medicine and Health Sciences, University of Antwerp, Antwerp, Belgium.

[3]Centre for Population, Family & Health, Department of Social Sciences, University of Antwerp, Antwerp, Belgium.

[4]Centre for Health Systems Research & Development, University of the Free State, Bloemfontein, South Africa.

¶ Both authors contributed equally to this paper.

(*Corresponding author: mmartens@itg.be)

Supplementary file 5. Interview Guide

1. Introduction

Introduction of the researcher(s)

- Thank
- Name & function/background of researcher
- Share researcher's contact details (e.g. business card)
- In case of two researchers: one asking questions, both taking notes

Introduction of SCUBY

- The **aim** of this research is to engage with key stakeholders at all levels, to identify opportunities and barriers to integrated chronic care at the macro level, to implement and support best practices on a larger scale. This research is linked to 2 larger projects (SCUBY and FAITH.be).
- **SCUBY** is a 4-year research project on the scaling up of integrated care for diabetes and hypertension through co-creation with key stakeholders, such as you ... We are studying the scaling up of integrated care for diabetes and hypertension, with special attention to vulnerable people.
- **FAITH.be** stands for 'Federated consortium for Appraisal of Integrated care Teams in Health in Belgium' and brings together faculties of 6 different universities (UA, Ugent, KUL, Ulg, UCL, VUB) for the evaluation of the pilot projects' integrated care for chronic sick.
- **Synergy with FAITH.be**: need for mapping macro context for integrated care
- **Key terms/focus on:** Integrated care for chronic diseases, diabetes, hypertension & vulnerable groups

About the interview:

- Aim of interview: gaining insight into your position in relation to integrated care and current policy initiatives and mapping of the macro context
- Duration of interview (max 60min)
- Ensure confidentiality (and anonymity in data analysis, without use of name)
- Informed consent: ask consent, sign form, and consent to record interview
- Questions?

Outline of interview (topics to cover)

- Policies on integrated chronic care (ICC)
- Barriers and facilitators (challenges & solutions)
- Stakeholders
- Remarks and conclusion

I. Warm up questions

On the position of the representative within his/her organization:

Q#1.1 - Can you briefly describe your position and role within your organization? [interest/power?]

On the organization:

Q#1.2 - Can you shortly describe the main goals and main activities of your organization? [resources!]

II. Policies on integrated chronic care (ICC)

General vision on integrated care
Q#2.1 - What is your **understanding** of integrated chronic care? What are key elements in your experience? [knowledge of the policy]
Q#2.2 - How is it relevant to diabetes and cardiovascular care? [knowledge of the policy]

Present policy map
We have prepared this timeline which shows policy trends in integrated care and related key policy documents. (Feedback? Is something missing?)
Q#2.3 - Which of these categories:
a) I strongly support it (S)
b) I somewhat support it (MS)
c) I do not support nor oppose it (N)
d) I somewhat oppose it (MO)
e) I strongly oppose it (O)
best describes **your opinion on:**

**1) Care trajectory?**	**2) 1st line reform in Fl?**	**3) Integreo?**
a) S<br>b) MS<br>c) N<br>d) MO<br>e) O	a) S<br>b) MS<br>c) N<br>d) MO<br>e) O	a) S<br>b) MS<br>c) N<br>d) MO<br>e) O

* *S=Support, MS=Moderate Support, N=Neutral, MO=Moderately Opposed, O=Opposed*
(Read the options and circle the answer given.) [position - self]

+ Why? Reasons of support? Why do you think it does/does not work?

Q#2.4 - What are **potential benefits of … to your organisation**? [interest]
- the care pathways (Zorgtrajecten)?
- the integreo projects ("Plan geïntegreerde zorg voor chronisch zieken")?
- the first line health reform in Flanders ("De herorganisatie van de eerste lijn in Vlaanderen")?

Q#2.5 - What are **potential disadvantages of … to your organisation**? [interest]
- the care pathways (Zorgtrajecten)?
- the integreo projects ("Plan geïntegreerde zorg voor chronisch zieken")?
- the first line health reform in Flanders ("De herorganisatie van de eerste lijn in Vlaanderen")?

Q#2.6 - What is **your role** in these initiatives? Do you have influence? Which means do you have available? [power/resources]

Q#2.7 - How do we move from the current system towards integrated chronic care in Belgium? Which **strategies** can be used? E.g. bundled payment, population management? Would you / your organisation take the initiative in this move? [leadership]

III. Barriers and challenges (but also facilitators & solutions)

POLICIES

Q#3.1 - Which/where are according to you the **barriers and challenges** for better care integration? [resources]

- In the political system?
- In the decision making processes?
- According to the division of responsibilities at different levels?
- In the health care financing system?
- Information technology?
- Available resources?
- Available competences?
  - Local managers/governance
  - Care providers
  - Patients
- Other aspects of health care system?

RESOURCES

Q#3.2 - What is the **main hurdle** and what can you do to optimize care? [resources]

IV. Stakeholders

STAKEHOLDERS

We would now like to ask you a few specific questions about your opinion regarding others' opinions of the implementation of ICC.

Q#4.1 - What are **the key stakeholders/organisations** to work with on ICC, in your view? Whose role is **central** in the achievement of ICC? Who should be **in charge** of which reforms? [alliance-leadership]

Q#4.2 - How would you describe your **collaboration** with or relation to the key stakeholders? How do they interact with other caregivers and social organisations? Who do you work closely with? [alliance]

Q#4.3 - What other organizations, departments within an organization, or persons do you think would **support** *[ICC]*? (Probe for MOH and non-MOH stakeholders) [position - others] What do you think these supporters would **gain** from *[ICC]*? [position - others] Which of these supporters would **take the initiative** to actively support *[ICC]*? [leadership]

Q#4.4 - What other organizations, departments within an organization, or persons do you think would **oppose** *[ICC]*? (Probe for MOH and non-MOH stakeholders) [position - others] What do you think these opponents would gain from preventing *[the ICC]*? Why would they be opposed? [position - others]

VULNERABLE PEOPLE

Q#4.5 - What are vulnerable populations in your view? How are they identified? [knowledge of the policy]

Briefly explain how we have defined vulnerable people in SCUBY.

multimorbide patiënten (=HT + T2D / HT+ other / T2D + other), patiënten over 65 en minderheidsgroepen (bv. etnisch & socio-economisch)

Q#4.6 - How do you reach vulnerable people? [power: ability to mobilize resources]

V. Concluding remarks & conclusion

To conclude:

Q#5.1 From a scale for 1 (no integration) to 10 (fully integrated), how far we are in the movement/transition towards integrated care – according to you? Why did you pick this score? How would you explain this?

Additional comments

- Do you have any additional remarks?
- Is there something that you think we didn't cover that is still relevant to this issue / topic? (Is there someone else you think we should talk to, that you can identify as a key stakeholder?)

Next steps

- E.g.: We'll be analysing the information you and others gave us and submitting a draft report in 3 months. I'll be happy to send you a copy to review (our findings) at that time, if you are interested.

Thank

Thank you for your time.

Share SCUBY brochure at the end.
