# Supplementary material for: Integration or Fragmentation of Health Care? Examining Policies and Politics in a Belgian Case Study
Source: Int J Health Policy Manag. 2021 Jul 6;11(9):1668–81. doi: 10.34172/ijhpm.2021.58 (PMC9808233; doi:10.34172/ijhpm.2021.58)
Supplement: Supplementary file 6 — Stakeholder Table With Scores (All Participants). [file ijhpm-11-1668-s006.pdf]

Article title: Integration or Fragmentation of Health Care? Examining Policies and Politics in a Belgian Case Study

Journal name: International Journal of Health Policy and Management (IJHPM)

Authors' information: Monika Martens[1,2]*¶, Katrien Danhieux[2]¶, Sara Van Belle[1], Edwin Wouters[3,4], Wim Van Damme[1], Roy Remmen[2], Sibyl Anthierens[2], Josefien Van Olmen[2]

[1]Department of Public Health, Institute of Tropical Medicine, Antwerp, Belgium.

[2]Department of Family Medicine and Population Health (FAMPOP), Faculty of Medicine and Health Sciences, University of Antwerp, Antwerp, Belgium.

[3]Centre for Population, Family & Health, Department of Social Sciences, University of Antwerp, Antwerp, Belgium.

[4]Centre for Health Systems Research & Development, University of the Free State, Bloemfontein, South Africa.

¶ Both authors contributed equally to this paper.

(*Corresponding author: mmartens@itg.be)

Supplementary file 6. Stakeholder Table With Scores (All Participants)

**Policies / Stakeholder**	**T2D care trajectory**			**National plan on integrated care for chronic patients**			**Primary Care Reform Flanders**		
	Position	Interest	Power	Position	Interest	Power	Position	Interest	Power
Rating method	S<br>MS<br>N<br>MO<br>O	High<br>Medium<br>Low	6) 3R+L<br>5) 3R−L<br>4) 2R+L<br>3) 2R−L<br>2) 1R+L<br>1) 1R−L	S<br>MS<br>N<br>MO<br>O	High<br>Medium<br>Low	6) 3R+L<br>5) 3R−L<br>4) 2R+L<br>3) 2R−L<br>2) 1R+L<br>1) 1R−L	S<br>MS<br>N<br>MO<br>O	High<br>Medium<br>Low	6) 3R+L<br>5) 3R−L<br>4) 2R+L<br>3) 2R−L<br>2) 1R+L<br>1) 1R−L
1. Federal Cabinet for PH	MO	Low	5) 3R−L	O	Low	5) 3R−L	N	Low	3) 2R−L
2. Federal administration (FOD)	N/D	Low	N/D	S	High	3) 2R−L	N	Low	1) 1R−L
3. Flemish Cabinet for PH	MO	Low	5) 3R−L	MO	Medium	5) 3R−L	S	High	6) 3R+L
4. Flemish administration (VAZG)	MO	Low	3) 2R−L	N	Low	3) 2R−L	S	High	5) 3R−L
5. Flemish cities and municipalities (VVSG)	N/A	N/A	N/A	N/D	Low	1) 1R−L	S	High	3) 2R−L
6. NIHDI	S	High	3) 2R−L	S	High	3) 2R−L	MS	Low	1) 1R−L
7. National Inter-mutualistic College (NIC)	N/D	N/D	N/D	MO	Medium	1) 1R−L	S	Medium	1) 1R−L
8. Sickness fund 1 (CM)	MS	Medium	3) 2R−L	MS	Medium	3) 2R−L	S	High	3) 2R−L
9. Sickness fund 2 (Socmut)	MS	Medium	3) 2R−L	MS	Medium	3) 2R−L	S	Medium	1) 1R−L
10. Medical syndicate 1 (DM)	S	High	3) 2R−L	MO	High	3) 2R−L	S	High	4) 2R+L
11. Medical syndicate 2 (ASGB)	S	High	4) 2R+L	MS	High	3) 2R−L	MS	High	1) 1R−L
12. Medical syndicate 3 (BVAS)	S	High	3) 2R−L	O	High	3) 2R−L	MO	High	2) 1R+L
13. General pharmaceutical association (APB)	MS	Low	1) 1R−L	MS	High	2) 1R+L	S	Medium	2) 1R+L
14. Care Network of hospitals and elderly care (Icuro)	MS	Medium	1) 1R−L	MS	Medium	3) 2R−L	MS	High	3) 2R−L
15. Dieticians association	MS	High	1) 1R−L	MS	Medium	1) 1R−L	S	Medium	2) 1R+L
16. Diabetes nurses (BVVDV)	S	High	2) 1R+L	N/A	N/A	N/A	N/A	N/A	N/A
17. Self-employed nurses (VBZV)	MS	High	1) 1R−L	MO	High	1) 1R−L	MO	High	1) 1R−L
18. Home care (Zorggezind)	N/A	Low	1) 1R−L	N/A	High	1) 1R−L	S	High	3) 2R−L
19. Salaried nurses association (WGK)	MS	High	1) 1R−L	MO	High	3) 2R−L	MS	High	4) 2R+L
20. Knowledge Centre for Health Care (KCE)	MS	Low	1) 1R−L	MS	Low	1) 1R−L	MS	Low	1) 1R−L

21. Academia	MS	Low	1) 1R−L	MS	Low	1) 1R−L	MS	Low	1) 1R−L
22. FAITH.be Research Consortium	MS	Low	1) 1R−L	S	High	3) 2R−L	N/D	Low	1) 1R−L
23. Flemish Patient Platform	MS	Medium	1) 1R−L	MS	High	3) 2R−L	S	High	4) 2R+L
24. Diabetes association (Diabetes Liga)	S	High	2) 1R+L	N/A	Low	1) 1R−L	N/A	Low	1) 1R−L

Notes:

(1) Scores in white are included in the analyses in this paper. Those (in grey boxes) with low interest and no power and medium interest and no power are excluded from the visual maps.

(2) N/A (not applicable) indicates that the interviewed stakeholder lacked knowledge about the policy in question (usually indicating their limited responsibility and involvement in the policy).

(3) N/D (no data) indicates that the policy was not extensively covered during the interview (the position was not made clear by the participant him/herself or by other stakeholders and could not be supported by additional consultation of grey literature).
